# Erythrocytosis in congenital heart defects: hints for diagnosis and therapy from a clinical case

**DOI:** 10.3389/fmed.2024.1419092

**Published:** 2024-08-12

**Authors:** Oscar Borsani, Marzia Varettoni, Giacomo Riccaboni, Elisa Rumi

**Affiliations:** ^1^Department of Molecular Medicine, University of Pavia, Pavia, Italy; ^2^Department of Hematology, Fondazione Istituto di Ricovero e Cura a Carattere Scientifico Policlinico San Matteo, Pavia, Italy

**Keywords:** erythrocytosis, congenital, heart, Eisenmenger, syndrome

## Abstract

Erythrocytosis is one of the most common abnormalities that clinical hematologists, general practitioners, and internal medicine specialists could have to face off in their routine clinical practice. While diagnostic criteria for primary erythrocytosis (i.e., polycythemia vera) are well known and characterized, there are several causes of secondary erythrocytosis that should be kept in mind to avoid misdiagnosis. Congenital heart defects are rarely cause of secondary erythrocytosis as they are normally recognized and treated at an early stage. Eisenmenger syndrome is a complex clinical syndrome that arise as consequence of an untreated congenital heart defect associated with large intracardiac shunt. The clinical picture of this syndrome usually includes a severe erythrocytosis that could tempt clinicians to start an intensive phlebotomy (or venesection) program. However, clinicians should be aware that erythrocytosis in Eisenmenger syndrome is a compensatory mechanism aimed at improving blood oxygen-carrying capacity; accordingly, phlebotomies should be reserved for those cases complaining hyperviscosity symptoms. Here we present a case of an adult female patient with Eisenmenger syndrome that has been evaluated because of severe and persistent erythrocytosis. In this case we present a step-by-step approach by which clinical hematologist could proceed to reach the definitive diagnosis. We will also provide some hints that could help clinicians when choosing the best treatment strategy to avoid unnecessary and potentially harmful procedures.

## 1 Introduction

Erythrocytosis commonly refers to a clinical condition characterized by a higher-than-normal red cell mass (RCM). Historically, isotope dilution methods has been considered the gold standard for the assessment of RCM (1); however, given the limited availability of this testing, hemoglobin (Hb) and hematocrit (Hct) values are commonly used as surrogate marker of RCM and completed replaced RCM calculation in the last edition of the WHO criteria for diagnosis of polycythemia vera (PV) (2). While WHO criteria clearly define specific Hb and Hct thresholds required for a diagnosis of PV (2), the most appropriate Hb and Hct thresholds to define a clinically significant erythrocytosis outside of PV are not universally recognized (3).

Whenever increased Hb and/or Hct values are detected, relative erythrocytosis due to plasma volume contraction (hemoconcentration) should be ruled out. Accordingly, signs and symptoms of dehydration should be considered (e.g., hypotension, xerostomia, headache, dizziness, confusion, oliguria, fatigue, thirst, and increased capillary nail refill time), and all causes of relative erythrocytosis should be excluded (e.g., diuretic use, gastrointestinal fluid losses, and alcohol or tobacco abuse) (4).

Once the presence of absolute erythrocytosis (i.e., due to a real increase in RCM) has been established, distinction between primary and secondary forms must be performed (Table 1).

**TABLE 1:** Pathophysiological classification of different causes of erythrocytosis.

Primary erythrocytosis	Secondary erythrocytosis
Congenital • EPO receptor mutations	Congenital • Mutations in proteins involved in oxygen-sensing pathways (e.g., mutations of HIF1A, VHL, and PHD) • High oxygen-affinity hemoglobin • Bisphosphoglycerate mutase deficiency
Acquired • Polycythemia vera	Acquired • Hypoxia-driven (chronic lung disease, right-to-left cardiopulmonary shunts, high-altitude habitat, tobacco use, carbon monoxide poisoning, sleep apnea syndrome, and renal artery stenosis) • Hypoxia-independent ° Pathologic EPO production (e.g., EPO-producing tumors) ° Exogenous EPO administration (drug related)

EPO, erythropoietin; HIF1α, hypoxia inducible factor 1 subunit alpha; VHL, Von Hippel-Lindau tumor suppressor; PHD, proline hydroxylases.

Primary erythrocytosis is characterized by an increased red-blood cells (RBC) production because of a congenital [i.e., erythropoietin (EPO) receptor mutation] (5) or acquired (i.e., PV) (2) bone marrow defect. In primary erythrocytosis, serum EPO levels are usually lower than normal because of negative feedback that high blood oxygen levels exert on EPO-producing renal cells.

In contrast, secondary erythrocytosis are usually EPO-driven and can be classified as congenital or acquired. Congenital secondary erythrocytosis may be due to the presences of high oxygen-affinity Hb (6), mutations in proteins involved in oxygen-sensing pathways (7–9) or because of bisphosphoglycerate mutase deficiency (10). Acquired secondary erythrocytosis are usually driven by increased levels of EPO because of hypoxia condition (endogenous adaptive production), pathologic production (EPO-producing tumors) or external administration (drug related) (11).

## 2 Cyanotic congenital heart diseases and adaptive mechanisms to chronic hypoxemia

Cyanotic congenital heart diseases (CCHD) are a heterogenous group of congenital heart malformations characterized by structural abnormalities of heart or intrathoracic great vessels that ultimately results in low oxygen blood level (hypoxemia).

The common mechanism that underlies hypoxemia in CCHD is the presence of anatomical abnormalities that allow deoxygenated blood (or at least a part of it) to bypass lungs and enter systemic circulation without being oxygenated, thus producing a mixture of oxygenated and unoxygenated blood that results in hypoxemia and systemic hypoxia. This long-standing hypoxemia is sensed by kidneys and causes an increased renal EPO production which, in turn, is responsible for erythrocytosis (12, 13).

Another adaptive mechanism that plays a role in chronic hypoxemia states consists in the slight rightward shift of the oxygen-Hb dissociation curve aimed at improving oxygen delivery to peripheral tissues by increasing its release from Hb (14). Indeed, it has been demonstrated that, in hypoxic conditions, RBC increase 2,3-bisphosphoglicerate (2,3-DPG) production by shunting its glycolytic precursor, 1,3-bisphosphoglicerate (1,3-BPG), to the Luebering–Rapoport pathway, in which a phosphoryl group is transferred from the C1 to C2 of 1,3-BPG by the enzyme bisphosphoglycerate mutase (15). Once generated, 2,3-BPG binds to Hb and produce a rightward shift of the oxygen-Hb dissociation curve, thus reducing the oxygen affinity of Hb (i.e., increasing Hb p50) and facilitating oxygen release from RBC to peripheral tissues (16).

## 3 Management of erythrocytosis in cyanotic congenital heart diseases

According to actual guidelines (17), patients affected with PV should be treated with phlebotomies and/or cytoreductive therapy to reach and maintain Hct value less than 45% in order to reduce the risk of thrombotic events associated with this myeloproliferative disease.

Secondary erythrocytosis may be clinically distinguished in “compensated erythrocytosis” or “decompensated erythrocytosis” according to erythrocytes indices, iron status and presence (or absence) of hyperviscosity symptoms (e.g., headache, visual disturbances, tinnitus, fatigue, dizziness, lightheadedness, and acral paresthesias).

In compensated erythrocytosis, chronic hypoxemia induces an appropriate increase in Hct level to reach a new equilibrium in which the higher RCM restores normal blood oxygen levels and allow normal tissues oxygenation. In these cases, iron metabolism is preserved and hyperviscosity symptoms are generally absent or mild.

On the other hand, decompensated erythrocytosis is characterized by the inability to reach a new equilibrium despite high Hct level. In these cases, dietary iron is usually insufficient to balance the physiological request and a negative iron balance is generally observed, producing an iron-deficiency erythrocytosis and, accordingly, microcytosis (12).

The Hct threshold value for phlebotomy is less defined for patients with secondary erythrocytosis. The British Society for Haematology recommends that phlebotomies should be provided to maintain a Hct level less than 55% and suggests to consider a lower Hct target whenever other cardiovascular risk factors are present or for those patients that reported previous thrombotic events (11).

Nowadays, there are no studies that define the optimal Hct level for patients with CCHD. According to the actual clinical practice, patients affected with CCHD are phlebotomized to maintain Hct lower than 65% or whenever hyperviscosity symptoms are reported (18). Phlebotomies cause a fall in RCM and, consequently, in serum viscosity. This, in turn, leads to a reduction of peripheral vascular resistances that cause an increase in stroke volume and cardiac output. The increased systemic blood flow leads in turn to an increased oxygen delivery to peripheral tissues, thus ameliorating hyperviscosity symptoms (19).

According to previous studies that reported a relation between high Hct values and an increased risk of thrombotic events (20) and to the well-known inverse relationship between cerebral blood flow and Hct level reported in patients with PV (21), phlebotomies are supposed to also reduce the risk of thrombotic strokes. However, studies failed to demonstrate the same thrombotic risk in patients with CCHD, thus arguing how phlebotomies should be performed in patients affected with CCHD (22). Indeed, it has been demonstrated that chronic phlebotomies lead to iron deficiency and consequently to the development of microcytosis. Microcytic RBCs are stiffer and less deformable than normal erythrocytes (23), thus leading to an increased risk of hyperviscosity symptoms and of cerebrovascular events (24, 25).

Therefore, it is noteworthy that chronic phlebotomies, while having a temporary beneficial effect in lowering Hct levels, could exacerbate hyperviscosity symptoms and cerebrovascular events by facilitating an iron-depletion status that leads to production of microcytic, rigid, and less-deformable erythrocytes.

## 4 Clinical case description

A 36-year-old female was evaluated at the Emergency Department of our hospital because of severe dyspnea of new onset.

**FIGURE 1:**
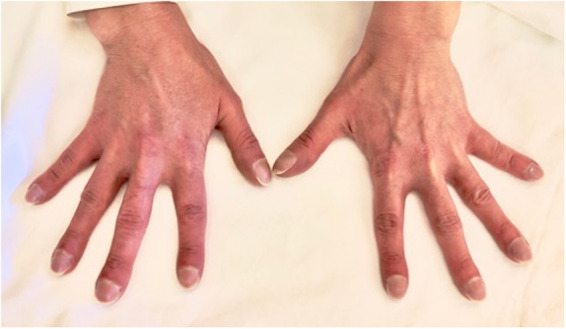
Digital clubbing presenting as bulbous uniform swelling of the soft tissue of the terminal phalanx of all digits with subsequent loss of the normal angle between the nail and nail bed.

Her past medical history was remarkable for an untreated perimembranous ventricular septal defects since her childhood. Physical examination revealed a central cyanosis and a symmetrical digital clubbing (Figure 1), a grade 2 systo-diastolic cardiac murmur and a symmetrical absence of lung sounds at both lung bases.

**FIGURE 2:**
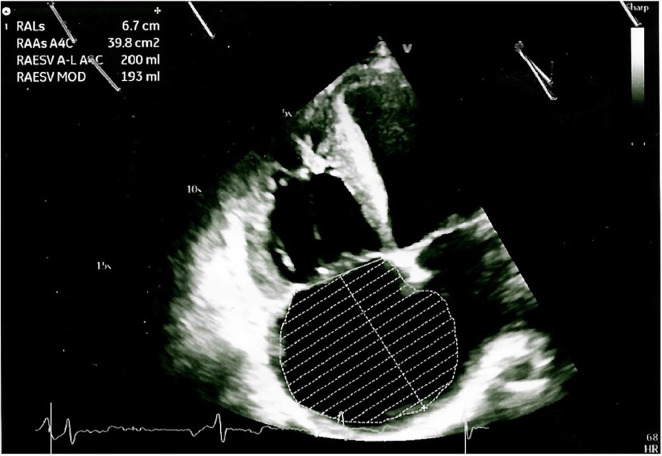
Doppler echocardiography showing cardiac signs of PAH, including right ventricular dilatation, severe tricuspid regurgitation, and a severe right atrial dilatation.

The arterial blood gas analysis performed at admission revealed a severe hypoxemia (pO2 30.4 mmHg) for which non-invasive oxygen administration was started. A Doppler echocardiography detected an increased pulmonary artery pressure and other signs of pulmonary arterial hypertension (PAH), including right ventricular dilatation, severe tricuspid regurgitation and a severe right atrial dilatation (Figure 2); it also revealed the well-known sub-aortic ventricular septal defect associated with a bidirectional shunt. A right heart catheterization was performed confirming a severe PAH.

The patient was diagnosed with Eisenmenger syndrome (ES).

A complete blood count revealed a marked erythrocytosis (hemoglobin 175 g/L, hematocrit 58.8%, leucocytes 6.13 × 10^9^/L, platelets 154 × 10^9^/L) for which hematology consultation was requested. A complete assessment of the erythrocytosis was performed, showing normal serum EPO level (9 mU/ml) and absence of V617F and exon 12 *JAK2* mutations.

These results confirmed the secondary nature of the erythrocytosis so that phlebotomies were not prescribed.

## 5 Eisenmenger syndrome: clinical management of erythrocytosis

Eisenmenger syndrome was first described by Victor Eisenmenger in 1897 as a condition characterized by PAH due to increased pulmonary vascular resistances that causes shunt at aorto-pulmonary, ventricular or atrial level (26).

This syndrome is the consequence of a CCHD associated with large, unrepaired atrial or ventricular septal defects that causes long-standing PAH and, consequently, reversion of the original left-to-right shunt to a right-to-left shunt (27). While high-income countries have drastically reduced the development of ES because of early diagnosis and surgical correction of CCHD (28), ES is still a clinical problem in middle- and low-income countries (29). Some cases of patients with long-term survival have been reported, however most of patients die before 40 years old (26).

The clinical picture of ES is characterized by chronic hypoxemia and heterogeneous multiorgan involvement.

Patients with ES experience variable long asymptomatic period after which they undergo to a progressive onset of exercise intolerance and exertion dyspnea. Other symptoms gradually emerge, including chest pain, syncope, heart failure, and rhythm disturbances (especially atrial fibrillation) (30). Sometimes, ES patients present with hemoptysis which could be a consequences of rupture of pulmonary artery, hemorrhage from pulmonary arteriole, pulmonary thromboembolism, coagulation alterations, or thrombocytopenia (30).

Most of physical signs that could be observed in patients affected with ES depends on the chronic hypoxemia state and include cyanosis, clubbing fingers and, whenever right ventricular hypertrophy is present, a parasternal lift. Examination of heart sounds reveals a systolic murmur due to tricuspid regurgitation and a fixed split of the S2 (30).

Patients presenting with hyperviscosity symptoms should be assessed to rule out dehydration or other causes. Brain imaging should be considered whenever neurological symptoms or signs are present to exclude the presence of a brain abscess, which is a common and potentially life-threatening complication in patients affected with ES (31, 32).

Presence of a chronic hypoxemia state leads to erythrocytosis which is a hallmark of this syndrome and is associated with multiple systemic involvement, including gout, renal dysfunction (which is associated with a worse survival), osteoarthropathy and gallstones formation (33, 34). Chronic tissue hypoxia observed in ES lead to a progressive impairment of renal function by both a direct effect of hypoxia on renal function and an indirect effect through secondary erythrocytosis and associated hyperviscosity, glomerulopathy, and interstitial and tubular damage (35).

According to the clinical distinction between compensated and decompensated erythrocytosis, secondary erythrocytosis observed in ES is usually decompensated and, consistently, iron deficiency is often present.

As stated before, iron deficiency typically found in secondary decompensated erythrocytosis is associated with increased manifestation of hyperviscosity symptoms and with a higher rate of thrombotic events (24, 25). Thus, iron supplementation could be safely administered in patients with ES and has been associated with an improvement of exercise tolerance and quality of life (36). However, it should be noted that in ES intestinal absorption of oral iron could be hampered and that, given the long “time to effect” of oral iron supplementation, intravenous iron administration should be considered (37). Patients that received iron supplementation should be carefully monitored for their iron status (by periodic evaluation of serum level of iron, transferrin and ferritin) in order to choose for how long iron supplementation should be continued.

According to the well-known role of iron deficiency in exacerbation of hyperviscosity symptoms (24, 25), therapeutic phlebotomies should not be considered in routine clinical practice (38, 39). Indeed, therapeutic phlebotomies should be provided only for patients with significantly increased Hb concentration (i.e., above 220 g/L) or Hct level (i.e., 65%) and for those patients presenting with hyperviscosity symptoms in which other etiologies (e.g., dehydration) have been ruled out (18). Whenever phlebotomy is performed, small volume of blood (ideally 200–250 ml) should be taken and a simultaneous intravenous fluid administration should be administered to avoid hemodynamic imbalance (18).

## 6 Conclusion

Secondary decompensated erythrocytosis could be a consequence of a CCHD. In these cases, iron deficiency plays a pivotal role in exacerbation of hyperviscosity symptoms and in increasing cerebrovascular thrombotic events (24, 25). ES is a complex syndrome caused by a CCHD with large unrepaired cardiac defect that ultimately leads to PAH and to a reversed right-to-left shunt (26, 27). Erythrocytosis due to chronic hypoxemia is a hallmark of ES and should be carefully assessed and managed. Venesections represent an appealing therapeutic option to counteract negative effects of erythrocytosis; however, it should be noted that iron deficiency could be worsened by periodic phlebotomies. For this reason, despite contrasting opinions about the role of therapeutic phlebotomies in ES, it is nowadays largely accepted that, whenever possible, this procedure should be avoided and reserved only for patients presenting with hyperviscosity symptoms or with significant high level of Hb or Hct (18).

## Ethics statement

Written informed consent was obtained from the individual(s) for the publication of any identifiable images or data included in this article.

## Author contributions

OB: Conceptualization, Data curation, Investigation, Validation, Writing – original draft, Writing – review & editing. MV: Data curation, Investigation, Writing – original draft, Writing – review & editing. GR: Data curation, Investigation, Writing – original draft, Writing – review & editing. ER: Conceptualization, Funding acquisition, Supervision, Validation, Writing – original draft, Writing – review & editing.

## References

[1] Pearson TC. Evaluation of diagnostic criteria in polycythemia vera. *Semin Hematol.* (2001) 38(Suppl. 2):21–4. 10.1016/s0037-1963(01)90136-2 11242598

[2] Khoury JD Solary E Abla O Akkari Y Alaggio R Apperley JF The 5th edition of the world health organization classification of haematolymphoid tumours: Myeloid and histiocytic/dendritic neoplasms. *Leukemia.* (2022) 36:1703–19. 10.1038/s41375-022-01613-1 35732831 PMC9252913

[3] Rumi E McMullin MF Harrison C Ellis MH Barzilai M Sarid N Facing erythrocytosis: Results of an international physician survey. *Am J Hematol.* (2019) 94:E225–7. 10.1002/ajh.25545 31148218 PMC8204407

[4] Mithoowani S Laureano M Crowther MA Hillis CM. Investigation and management of erythrocytosis. *CMAJ.* (2020) 192:E913–8. 32778603 10.1503/cmaj.191587 PMC7829024

[5] Petersen KB Hokland P Petersen GB Nyvold CG. Erythropoietin receptor defect: A cause of primary polycythaemia. *Br J Haematol.* (2004) 125:537–8. 15142125 10.1111/j.1365-2141.2004.04931.x

[6] Percy MJ Butt NN Crotty GM Drummond MW Harrison C Jones GL Identification of high oxygen affinity hemoglobin variants in the investigation of patients with erythrocytosis. *Haematologica.* (2009) 94:1321–2. 19734427 10.3324/haematol.2009.008037 PMC2738729

[7] Ang SO Chen H Hirota K Gordeuk VR Jelinek J Guan Y Disruption of oxygen homeostasis underlies congenital Chuvash polycythemia. *Nat Genet.* (2002) 32:614–21. 10.1038/ng1019 12415268

[8] Percy MJ Zhao Q Flores A Harrison C Lappin TR Maxwell PH A family with erythrocytosis establishes a role for prolyl hydroxylase domain protein 2 in oxygen homeostasis. *Proc Natl Acad Sci USA.* (2006) 103:654–9. 16407130 10.1073/pnas.0508423103 PMC1334658

[9] Percy MJ Furlow PW Lucas GS Li X Lappin TR McMullin MF A gain-of-function mutation in the HIF2A gene in familial erythrocytosis. *N Engl J Med.* (2008) 358:162–8. 18184961 10.1056/NEJMoa073123 PMC2295209

[10] Rosa R Prehu MO Beuzard Y Rosa J. The first case of a complete deficiency of diphosphoglycerate mutase in human erythrocytes. *J Clin Invest.* (1978) 62:907–15. 152321 10.1172/JCI109218 PMC371847

[11] McMullin MFF Mead AJ Ali S Cargo C Chen F Ewing J A guideline for the management of specific situations in polycythaemia vera and secondary erythrocytosis: A British society for haematology guideline. *Br J Haematol.* (2019) 184:161–75. 10.1111/bjh.15647 30426472 PMC6519221

[12] Rosove MH Perloff JK Hocking WG Child JS Canobbio MM Skorton DJ. Chronic hypoxaemia and decompensated erythrocytosis in cyanotic congenital heart disease. *Lancet.* (1986) 2:313–5. 10.1016/s0140-6736(86)90005-x 2874330

[13] Tyndall MR Teitel DF Lutin WA Clemons GK Dallman PR. Serum erythropoietin levels in patients with congenital heart disease. *J Pediatr.* (1987) 110:538–44. 3559801 10.1016/s0022-3476(87)80544-9

[14] Berman W Wood SC Yabek SM Dillon T Fripp RR Burstein R. Systemic oxygen transport in patients with congenital heart disease. *Circulation.* (1987) 75:360–8. 3802439 10.1161/01.cir.75.2.360

[15] Rapoport I Berger H Elsner R Rapoport SPH-. dependent changes of 2,3-bisphosphoglycerate in human red cells during transitional and steady states in vitro. *Eur J Biochem.* (1977) 73:421–7. 10.1111/j.1432-1033.1977.tb11333.x 14829

[16] Sasaki R Chiba H. Role and induction of 2,3-bisphosphoglycerate synthase. *Mol Cell Biochem.* (1983) 53-54:247–56. 10.1007/BF00225257 6312283

[17] Barbui T Passamonti F Accorsi P Pane F Vannucchi AM Velati C Evidence– and consensus-based recommendations for phlebotomy in polycythemia vera. *Leukemia.* (2018) 32:2077–81. 10.1038/s41375-018-0199-5 29955128

[18] Arvanitaki A Gatzoulis MA Opotowsky AR Khairy P Dimopoulos K Diller GP Eisenmenger syndrome: JACC state-of-the-art review. *J Am Coll Cardiol.* (2022) 79:1183–98. 35331414 10.1016/j.jacc.2022.01.022

[19] Oldershaw PJ Sutton MG. Haemodynamic effects of haematocrit reduction in patients with polycythaemia secondary to cyanotic congenital heart disease. *Br Heart J.* (1980) 44:584–8. 10.1136/hrt.44.5.584 7437201 PMC482448

[20] Tohgi H Yamanouchi H Murakami M Kameyama M. Importance of the hematocrit as a risk factor in cerebral infarction. *Stroke.* (1978) 9:369–74. 675749 10.1161/01.str.9.4.369

[21] Pearson TC. Rheology of the absolute polycythaemias. *Baillieres Clin Haematol.* (1987) 1:637–64. 3327560 10.1016/s0950-3536(87)80019-7

[22] Perloff JK Marelli AJ Miner PD. Risk of stroke in adults with cyanotic congenital heart disease. *Circulation.* (1993) 87:1954–9. 8504509 10.1161/01.cir.87.6.1954

[23] Linderkamp O Klose HJ Betke K Brodherr-Heberlein S Bühlmeyer K Kelson S Increased blood viscosity in patients with cyanotic congenital heart disease and iron deficiency. *J Pediatr.* (1979) 95:567–9. 480036 10.1016/s0022-3476(79)80770-2

[24] Ammash N Warnes CA. Cerebrovascular events in adult patients with cyanotic congenital heart disease. *J Am Coll Cardiol.* (1996) 28:768–72. 8772770 10.1016/0735-1097(96)00196-9

[25] Cottrill CM Kaplan S. Cerebral vascular accidents in cyanotic congenital heart disease. *Am J Dis Child.* (1973) 125:484–7. 4699887 10.1001/archpedi.1973.04160040010003

[26] Wood P. The Eisenmenger syndrome or pulmonary hypertension with reversed central shunt. *Br Med J.* (1958) 2:755–62. 13572894 10.1136/bmj.2.5099.755 PMC2026272

[27] Arvanitaki A Giannakoulas G Baumgartner H Lammers AE. Eisenmenger syndrome: Diagnosis, prognosis and clinical management. *Heart.* (2020) 106:1638–45. 32690623 10.1136/heartjnl-2020-316665

[28] Chaix MA Gatzoulis MA Diller GP Khairy P Oechslin EN. Eisenmenger syndrome: A multisystem disorder-do not destabilize the balanced but fragile physiology. *Can J Cardiol.* (2019) 35:1664–74. 10.1016/j.cjca.2019.10.002 31813503

[29] Kempny A Dimopoulos K Gatzoulis MA. Declining incidence and prevalence of Eisenmenger syndrome in the developed world: A triumph of modern medicine. *Heart.* (2017) 103:1313–4. 10.1136/heartjnl-2017-311396 28566473

[30] Kaemmerer H Mebus S Schulze-Neick I Eicken A Trindade PT Hager A The adult patient with Eisenmenger syndrome: A medical update after Dana point part I: Epidemiology, clinical aspects and diagnostic options. *Curr Cardiol Rev.* (2010) 6:343–55. 10.2174/157340310793566154 22043211 PMC3083816

[31] Daliento L Somerville J Presbitero P Menti L Brach-Prever S Rizzoli G Eisenmenger syndrome. Factors relating to deterioration and death. *Eur Heart J.* (1998) 19:1845–55. 10.1053/euhj.1998.1046 9886728

[32] Hjortshøj CMS Kempny A Jensen AS Sørensen K Nagy E Dellborg M Past and current cause-specific mortality in Eisenmenger syndrome. *Eur Heart J.* (2017) 38:2060–7. 10.1093/eurheartj/ehx201 28430906

[33] Dimopoulos K Diller GP Koltsida E Pijuan-Domenech A Papadopoulou SA Babu-Narayan SV Prevalence, predictors, and prognostic value of renal dysfunction in adults with congenital heart disease. *Circulation.* (2008) 117:2320–8. 18443238 10.1161/CIRCULATIONAHA.107.734921

[34] Martínez-Lavín M Amigo MC Castillejos G Padilla L Vintimilla F. Coexistent gout and hypertrophic osteoarthropathy in patients with cyanotic heart disease. *J Rheumatol.* (1984) 11:832–4. 6520838

[35] Flanagan MF Hourihan M Keane JF. Incidence of renal dysfunction in adults with cyanotic congenital heart disease. *Am J Cardiol.* (1991) 68:403–6. 1858686 10.1016/0002-9149(91)90842-9

[36] Tay EL Peset A Papaphylactou M Inuzuka R Alonso-Gonzalez R Giannakoulas G Replacement therapy for iron deficiency improves exercise capacity and quality of life in patients with cyanotic congenital heart disease and/or the Eisenmenger syndrome. *Int J Cardiol.* (2011) 151:307–12. 20580108 10.1016/j.ijcard.2010.05.066

[37] Blanche C Alonso-Gonzalez R Uribarri A Kempny A Swan L Price L Use of intravenous iron in cyanotic patients with congenital heart disease and/or pulmonary hypertension. *Int J Cardiol.* (2018) 267:79–83. 29807779 10.1016/j.ijcard.2018.05.062

[38] Condliffe R. Erythrocytosis and iron status in Eisenmenger syndrome: An illustrative case study. *J Congen Cardiol.* (2020) 4:11.

[39] Diller GP Lammers AE Oechslin E. Treatment of adults with Eisenmenger syndrome-state of the art in the 21st century: A short overview. *Cardiovasc Diagn Ther.* (2021) 11:1190–9. 10.21037/cdt-21-135 34527543 PMC8410485

